# Annotations

**Published:** 1896-04-18

**Authors:** 


### Annotations.

### Dr. Kingsbury and Professional Advertising.

Dr. Kingsbury, of Blackpool, has sued the *British Medical Journal* for damages for an alleged libel, and has secured a verdict against the *Journal*, carrying damages to the amount of £150, and costs. A full report of the trial has been forwarded to us, together with a number of handbills and other printed matter, showing how extensively a certain section of the profession is advertised by charitable and commercial agencies, with or without previous consent gained. The subject is both "fervent" and important, and we have made it our business to read the report of the trial, and to study the handbills, &c., with which we have been favoured. Two or three things remain in the mind after such a study, with a persistence and a prominence which are by no means agreeable. The first is that though everybody is convinced that professional advertising is a very wicked thing, nobody seems to be able to say exactly what professional advertising is. As one medical witness amusingly put it at the trial, nobody can tell us where "lamb ends and mutton begins." Another point is, that though many professional witnesses were convinced that every kind of publication of a medical neighbour's name was "advertising," no sort of publication of their own names had any tincture of advertising about it. A "lawyer" summing up the case as it appears to the legal mind is of opinion that the present chaotic condition of things cannot but be eminently satisfactory to the legal profession, because it obviously gives promise of litigation and "costs" without end. To use his own words, "so long as the ruling authorities of the medical profession are unable or unwilling to define what is meant by advertising the lawyers have no cause to complain." And that is manifest. But have the doctors, and has the public, no cause to complain? The medical profession never appeared to greater disadvantage, or gave more justifiable cause for legal and general "scoffing" than it did at the Kingsbury v. Hart trial. A number of "eminent" London medical men gave expert testimony in favour of Mr. Hart and the *British Medical Journal*; a similar number of equally "eminent" Manchester medical men gave equally expert and diametrically opposite testimony in favour of Dr. Kingsbury. A non-expert jury decided the point between them in favour of Dr. Kingsbury and against Mr. Hart and the *Journal*. So far as the profession is concerned the expert testimony and the trial decided nothing at all; and chaos, as before, still reigns supreme. If this be not a thing for the enemy to laugh at, and for doctors to be ashamed of, what is it?

### The Small-pox at Gloucester.

The outbreak of small-pox at Gloucester is one of those lessons in epidemiology whose teaching extends far beyond the mere controversy between vaccinators and non-vaccinators. The first and most important point is, that we receive from it fresh assurance—if assurance were needed—that there is no change in the nature and character of the disease with which we have to deal; that the small-pox of to-day is the same as that of a hundred and fifty years ago; and that, notwithstanding all that has so often been said to the contrary, if it were once let loose upon us, and if the barriers which have been opposed to its progress were removed, we should have again to deal with the same frightful malady which devastated and disfigured the nation during the last century. Of the patients attacked more than one in every five has succumbed. As to its prevalence, we can best judge by comparison with a known standard such as London. We hear outcry enough about the prevalence of scarlet fever and diphtheria and measles in London; but if all the zymotic diseases of London are put together, including diarrhœa, which carries off more than any of them, they do not altogether kill as large a proportion of the population, even during the whole year, as have been carried off in Gloucester by this short outbreak of this one disease. The importance of vaccination, and the desirability of submitting to the inconvenience which it entails, must largely hinge upon the view we take of the danger which it is meant to obviate, and this outbreak at Gloucester has, at least, served to show how great this danger is. On the other hand, it shows how imperfect is much of the vaccination which has been commonly accepted as efficient. It is impossible to foretell what the Vaccination Commission may recommend as to compulsion, but we may feel quite sure that they will urge that means shall be taken to greatly improve the character of such vaccination as is performed

### Medical Aid Societies and Midland Doctors.

The members of the British Medical Association of Birmingham are making some sensible efforts to check the abuses introduced into medical practice by medical aid societies. They have passed a resolution which cuts off the medical advisers of those societies from at least a certain amount of professional recognition, to wit, the recognition of those members of the profession who are loyal members of the B.M.A. It may be hoped that the effect of this decided action on the part of the local members of the B.M.A. will be to induce all the medical men of the neighbourhood who respect their profession and themselves to take up the same position. We are not among those who stand for narrow and unwritten rules of professional etiquette, if they happen to be manifestly contrary to common sense and the reasonable liberty of a man of full age. But all the more, because we insist upon the largest possible amount of liberty which is compatible with professional honour, do we stand resolutely for a reasonable discipline in the profession. Every organisation of men must have rules, and must loyally live by them. Where there are no rules there can be no organisation, and no corporate life, and therefore none of the manifold and inestimable advantages which spring from organisation and corporate life. The evil doings of medical aid societies are too well known to need more than bare mention. Among other things they employ paid canvassers to steal patients from all other classes of men except the doctors of their own societies. This one thing alone is quite sufficient to justify any medical man, other than a medical aid doctor, in refusing to have anything whatever to do with the dishonourable person who, without any excuse whatever, tries, by means of a third party, to steal away his livelihood. It is just as proper for the ordinary doctor to do his best to put an end to such underhand practices as it is for the patriotic soldier to defend his native shores against a foreign enemy. The Birmingham branch of the B.M.A. has our fullest sympathy in its endeavours to protect the legitimate interests of honourable practitioners.

